# Dynamically Cross-Linked Granular Hydrogels for 3D Printing and Therapeutic Delivery

**DOI:** 10.1021/acsabm.3c00337

**Published:** 2023-08-16

**Authors:** Hung-Pang Lee, Ryan Davis, Ting-Ching Wang, Kaivalya A. Deo, Kathy Xiao Cai, Daniel L. Alge, Tanmay P. Lele, Akhilesh K. Gaharwar

**Affiliations:** † Biomedical Engineering, College of Engineering, Texas A&M University, College Station, Texas 77843, United States; ‡ Chemical Engineering, College of Engineering, Texas A&M University, College Station, Texas 77843, United States; § Material Science and Engineering, College of Engineering, Texas A&M University, College Station, Texas 77843, United States; ∥ Interdisciplinary Graduate Program in Genetics & Genomics, Texas A&M University, College Station, Texas 77843, United States; ⊥ Center for Remote Health Technologies and Systems, Texas A&M University, College Station, Texas 77843, United States

**Keywords:** biomaterials, microgels, drug delivery, injectable, regenerative medicine

## Abstract

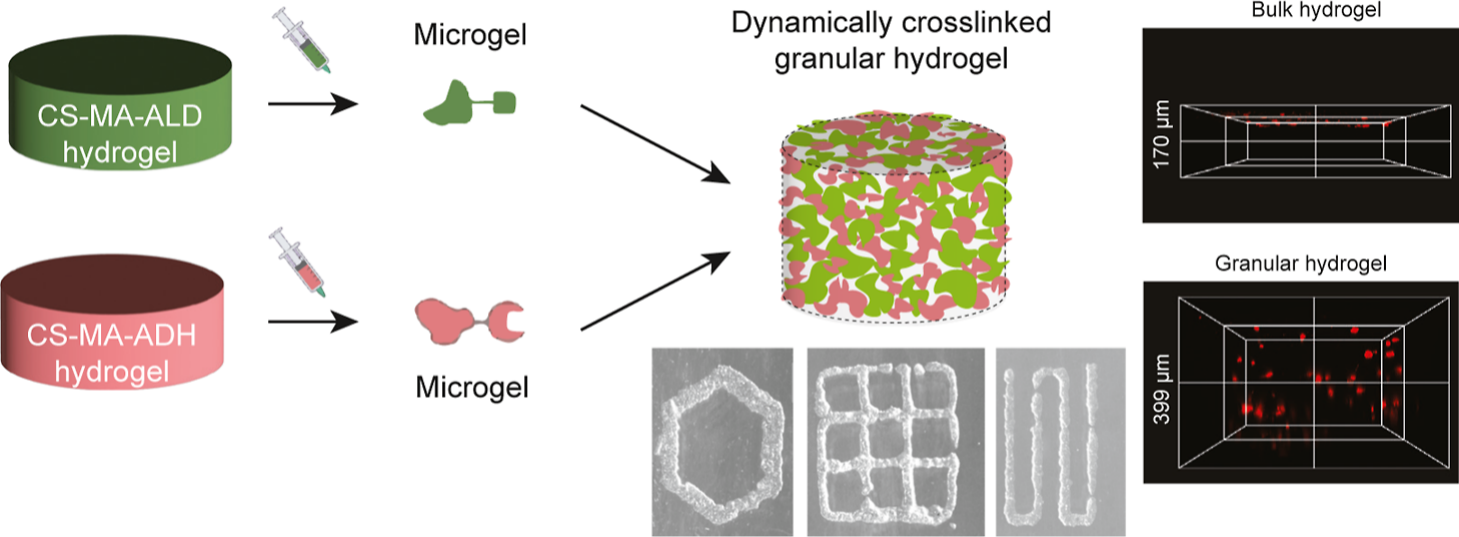

Granular hydrogels have recently emerged as promising biomaterials for tissue engineering and 3D-printing applications, addressing the limitations of bulk hydrogels while exhibiting desirable properties such as injectability and high porosity. However, their structural stability can be improved with post-injection interparticle cross-linking. In this study, we developed granular hydrogels with interparticle cross-linking through reversible and dynamic covalent bonds. We fragmented photo-cross-linked bulk hydrogels to produce aldehyde or hydrazide-functionalized microgels using chondroitin sulfate. Mixing these microgels facilitated interparticle cross-linking through reversible hydrazone bonds, providing shear-thinning and self-healing properties for injectability and 3D printing. The resulting granular hydrogels displayed high mechanical stability without the need for secondary cross-linking. Furthermore, the porosity and sustained release of growth factors from these hydrogels synergistically enhanced cell recruitment. Our study highlights the potential of reversible interparticle cross-linking for designing injectable and 3D printable therapeutic delivery scaffolds using granular hydrogels. Overall, our study highlights the potential of reversible interparticle cross-linking to improve the structural stability of granular hydrogels, making them an effective biomaterial for use in tissue engineering and 3D-printing applications.

## 1 Introduction

Hydrogels are attractive for a wide range of biomedical applications, including tissue engineering and therapeutic delivery, due to their biocompatibility and high tunability.^1−7^ However, conventional bulk hydrogels exhibit a nanoporous mesh, which limits cell infiltration and molecular transport. To address this limitation, granular hydrogels have emerged as useful tools in biomedical engineering.^8−10^ Granular hydrogels are typically formed by fabricating micro-sized gel particles using either top-down or bottom-up synthesis approaches and then packing them together through chemical or physical interactions. Top-down approaches involve the fragmentation of bulk hydrogels using techniques such as sonication or grinding,^11^ while bottom-up approaches involve the assembly of nano- or micro-building blocks to form 3D structures.^12,13^ Once the micro-sized gel particles are formed, they can be packed together through chemical^14,15^ or physical interactions.^11,16−19^ After packing, these granular hydrogels possess an interconnected microscale porosity that can be leveraged for a range of biomedical applications. For example, the increased pore size and interconnectivity allow for efficient nutrient transport as well as enhanced cell invasion,^20^ proliferation, and tissue formation.^21^

Inherently, granular hydrogels exhibit a strain-yielding property, indicating that high shear forces can trigger their disintegration into individual microgels during the injection.^22^ This trait parallels the behavior of Bingham fluids, where the substance maintains a solid-like state until reaching a specific yield point, after which it shifts to a fluid-like flow. For granular hydrogels, this enables a smooth, fluid-like flow upon injection, while simultaneously maintaining structural stability under less intense shear forces. Granular hydrogels are formed through a process known as jamming when external forces are applied to pack microgels *via* centrifugation or vacuum filtration.^23,24^ While the lack of complexity in these methods is advantageous, they are inefficient and highly unstable upon the removal of the external force. Therefore, microgels are often modified with functional groups to facilitate physical or chemical bonding between individual microgels and stabilize the structure.

Physical cross-linking facilitates interparticle cross-linking due to its spontaneous, reversible nature that provides shear-thinning, strain-yielding, and self-healing properties.^25−27^ Hydrogen bonding,^28^ host–guest interactions,^16^ and electrostatic interactions^29^ have each been used to form physically cross-linked granular hydrogels. Host–guest interactions based on cyclodextrin and adamantane have been used for polyethylene glycol or hyaluronic acid-based granular hydrogels, which have exhibited shear-thinning behavior and rapid cell invasion into the scaffolds.^22,27^ Chitosan- and gelatin-based microgels have also been used to form granular hydrogels by interparticle electrostatic interactions for 3D printing and tissue engineering.^30^ However, granular hydrogels formed by physical cross-linking often have poor mechanical stability.

Various covalent cross-linking strategies also have been employed to enhance the stability of granular hydrogels. For example, enzyme-triggered interparticle cross-linking using transglutaminase peptides annealed microgel building blocks by the addition of factor XIIIa.^8,31^ Clickable functional groups of azide and alkyne on the surface of microgels were also used for annealing microgel scaffolds, requiring no cross-linking reagents before injection.^25^ A slurry of these microgel building blocks can be delivered *via* syringe application before scaffold annealing, which could reinforce the stability of microgel scaffolds but also might limit operation time. Moreover, to have precise control over gelation time, photochemically annealed granular hydrogels with a thiol–ene reaction as secondary cross-linking are also often used for bioprinting and injectable hydrogel applications.^17,32^ However, these methods often rely on additional procedures or reagents to trigger cross-linking, which would complicate and limit the broad use of granular hydrogels in biomedical applications.

To reinforce injectable granular hydrogels, dynamic covalent bonds are introduced to cross-link microgels, such as boronate ester and hydrazone bonds.^33,34^ Both these bonds are reversible, even in mild conditions, which is very useful for providing self-healing properties to hydrogel materials.^33,35^ In this study, we used hydrazone bonds to facilitate interparticle cross-linking between photo-cross-linked microgel fragments, forming a granular hydrogel with efficient shear-thinning and self-healing properties. Relative to oxime, the higher reversibility of hydrazone bonds leads to improved injectability;^36^ relative to imine, the faster reaction kinetics of hydrazone bonds foster efficient cross-linking to stabilize granular hydrogels after injection. The base materials were chondroitin sulfate (CS), which was functionalized with methacrylate groups (CS-MA), followed by separate functionalization of CS-MA with an either aldehyde (CS-MA-ALD) or hydrazide (CS-MA-ADH) groups. To form the granular hydrogels, bulk hydrogels were first fabricated and extruded to facilitate the fragmentation of microgels. The aldehyde and hydrazide-containing microgels were mixed and jammed using vacuum filtration to form dynamically cross-linked granular hydrogels.

In this study, the structural, mechanical, and viscoelastic properties of the dynamically cross-linked granular hydrogels were characterized and compared to a control group without interparticle cross-linking. Subsequently, the injectability and self-healing properties of dynamically cross-linked granular hydrogels were assessed. Moreover, CS was selected due to its ability to absorb charged proteins and growth factors, such as transforming growth factor β (TGF-β)^37^ and platelet-derived growth factor-BB (PDGF-BB).^38^ The ability of the granular hydrogels to load and release biomolecules *in vitro* was investigated.^39^ The study showed the development of a tailored granular hydrogel that can be easily administered without the need for post-injection cross-linking. The granular hydrogel exhibited high porosity and sustained protein delivery, making it a promising candidate for applications as injectable and 3D-printed tissue scaffolds for tissue regeneration and cell recruitment.

## 2 Materials and Methods

### 2.1 Materials

CS and sodium metaperiodate were purchased from Alfa Aesar. Methacrylic anhydride, Irgacure 2959, chondroitinase ABC, and fluorescein isothiocyanate (FITC)–dextran were purchased from Sigma-Aldrich. Adipic acid dihydrazide, *N*-hydroxysuccinimide (NHS), and 1-(3-dimethylaminopropyl)-3-ethylcarbodiimide hydrochloride (EDAC–HCl) were purchased from VWR. Rhodamine–BSA and FITC–BSA were purchased from Rockland. Platelet-derived growth factor-BB was purchased from PeproTech.

### 2.2 Methacrylation of CS (CS-MA)

CS-MA synthesis was adapted from previously described protocols.^40^ Briefly, 2 g of CS was dissolved in 100 mL of deionized (DI) water, and the solution was adjusted to 4 °C and a pH of 8.5. Subsequently, 4.5 mL of methacrylic anhydride was added slowly, and the pH was maintained in the range of 7.5–8.5 for 3 h. This was followed by the second addition of 4.5 mL of methacrylic anhydride and further maintenance of the pH for 3 additional hours. The reaction was allowed to proceed overnight stirring at 700 rpm. The product was dialyzed for 7 days in DI water, frozen at −80 °C, and lyophilized for 5 days. The polymer was stored at −80 °C until further use.

### 2.3 Preparation of Aldehyde-Modified CS-MA (CS-MA-ALD) and Hydrazide-Modified CS-MA (CS-MA-ADH)

CS-MA-ALD synthesis was adapted from previously described protocols.^41^ Briefly, 500 mg of CS-MA was dissolved in 50 mL of DI water. Sodium metaperiodate (135 mg) was then added to the beaker. The reaction was allowed to proceed for 24 h in the dark. To quench the reaction, 1 mL of ethylene glycol was added to the beaker and stirred for 1 h. The product was dialyzed for 3 days in DI water. CS-MA-ADH synthesis was adapted from previously described protocols. Briefly, 800 mg of CS-MA was dissolved in 200 mL of DI water. ADH (7.04 g) was added to the beaker, and the pH was adjusted to 5.0 using 1 M hydrochloric acid. EDC–HCl (3.2 g) and NHS (460 mg) were added to the beaker, and the pH was maintained at 5.0 for 2 h. The reaction proceeded for 8 h, after which the product was dialyzed for 1 day in 0.1 M sodium chloride, followed by dialysis for 3 days in DI water. The two types of polymers were lyophilized and stored at −80 °C until further use. Lyophilized polymers were dissolved in deuterium oxide (D_2_O) at a concentration of 10 mg/mL and analyzed using ^1^H NMR (AVANCE NEO 400) to confirm the presence of functional groups and determine the degree of modification. To better quantify the aldehyde group, *t*-butyl carbazate and sodium cyanoborohydride were first reacted with CS-MA-ALD.^42^

### 2.4 Fabrication of Microgels and Granular Hydrogels

CS-MA, CS-MA-ADH, and CS-MA-ALD were dissolved in PBS at 5 wt % with an addition of 0.1 wt % I2959. To cross-link the bulk hydrogel, 1 mL of precursor solution was added to a 3 mL syringe and exposed to UV light (320–500 nm, 30 mW/cm^2^) for 10 min. To produce microgels, an extrusion fragmentation method was used. The bulk hydrogel was extruded through a series of syringe needles (18G, 23G, 27G, and 30G). To reduce the force required to extrude the hydrogel, 1 mL of PBS was added to the syringe after extrusion through the 18G needle. The microgels were then washed and centrifuged in PBS to remove any unreacted polymer. The microgels were then lyophilized and stored at −80 °C until further use. To form the granular hydrogels with dynamic covalent cross-linking, lyophilized CS-MA-ALD and CS-MA-ADH microgels were mixed at a 1:1 ratio, and the mixture was hydrated to 5 wt % in PBS. To form the CS-MA granular hydrogels as a control, lyophilized CS-MA microgels were hydrated to 5 wt % in PBS. After 15 min, these microgels were jammed using vacuum filtration. Specifically, a 350 mL Buchner funnel was placed inside a 500 mL volumetric flask, and a sheet of grade 415 filter paper (9 cm) was placed on top. The vacuum was generated by the vacuum aspiration pump, and microgel particles were placed on the filter paper to remove excess PBS.

### 2.5 Visualization of Microgel Fragments and Porosity

To visualize the granular hydrogels, 1 mg/mL FITC–dextran (2 MDa) was added to the CS-MA and CS-MA-ALD solutions, and 25 μg/mL rhodamine–BSA was added to the CS-MA-ADH solution before cross-linking. Fluorescent microgels were then produced by extrusion fragmentation, mixed in a 1:1 ratio, and jammed by vacuum-driven filtration. An inverted confocal microscope (Olympus FV 3000) fitted with a 10× or 20× objective lens was used to visualize the fluorescent microgels. To characterize the porosity of the microgel assemblies, the granular hydrogels were prepared as previously described and suspended in 1 mg/mL FITC–dextran, jammed by vacuum-driven filtration, and imaged on a confocal fluorescence microscope. Volumetric stacks 1272 μm × 1272 μm × 376 μm were taken at randomly selected regions with a *z*-spacing of 4 μm between successive stack slices. 26 images for each group were selected for final analysis. Pores were then analyzed using ImageJ software.

### 2.6 Degradation Testing

Granular hydrogel samples were prepared, weighed, and submerged in 1 mL of PBS or 0.05 U/mL chondroitinase ABC. The samples were incubated at 37 °C and at specific time points, certain samples were removed from the solution and weighed again to determine the remaining mass.

### 2.7 Compression Testing

Granular hydrogels were jammed into a PDMS mold (diameter = 6 mm) to form cylindrical samples. Mechanical testing using an ADMET eXpert 7600 system (ADMET, Inc., Norwood, MA, USA) with an attached load cell of 25 lb was performed to determine the compressive modulus, failure stress, and failure strain of each sample. Granular hydrogels were compressed to 30% strain at a rate of 1 mm/min, and the compressive moduli were calculated as the linear slope of the stress–strain curve from 0 to 20% strain. For a quantitative assessment of self-healing, compression testing was first performed on a set of granular hydrogel samples to determine the compressive modulus. Next, the samples were jammed into a rubber mold, fragmented using a pipette tip, and incubated overnight at 37 °C in PBS to heal. The compressive modulus of the recovered gels was then measured and compared to the initial value to determine the recovery of mechanical properties after healing.

### 2.8 Rheological Characterization

Rheological properties of the granular hydrogels were assessed using an oscillatory stress-controlled rheometer (Discovery HR-2, TA Instruments) equipped with a 20 mm parallel steel plate geometry and a 4 mm gap. Shear-rate sweeps were performed (0.01–1000 s^–1^) to assess the shear-thinning behavior of the granular hydrogels. Strain rate sweeps (0.1–500%) were used to determine the yield strain of the granular hydrogels. Shear stress sweeps (0.1 to 1000 Pa; GH or 5000 Pa; DCGH) were used to determine the yield stress of the granular hydrogels. Shear recovery was performed to characterize the reduction and recovery of the elastic properties of the granular hydrogels in response to periodically alternating strains of 1 and 100%.

### 2.9 Injection Force Quantification

The forces required to inject the granular hydrogels through various needles were analyzed with a mechanical tester using an ADMET eXpert 7600 system (ADMET, Inc., Norwood, MA, USA) with an attached load cell of 25 lb. Granular hydrogels were loaded into a 1 mL syringe which was placed into a 3D-printed stand that allowed the compression plate to apply an even force to the syringe plunger and extrude the gel. The injection rate was 2 mL/h, and the load generated was tracked over time. These tests were performed for 40 s. The peak force for each needle was identified as the highest load value reached during testing.

### 2.10 3D-Printing, Modeling, and Fidelity Quantification

Computational flow modeling was performed using ANSYS 2022. The power law model was fit to the results of rheological shear-rate sweeps to determine the power law index (*n*) and consistency factor (*K*), which were used as input variables in software. The maximum and minimum viscosity from the shear-rate sweeps were also used as input variables, and simulations were performed on a Solidworks model of a 22-gauge nozzle to determine velocity and strain rate gradients. The 3D printing was performed on a modified Anet A8 printer with screw-based extrusion. The 3D model designs were created in Solidworks 2020 and converted to STL files for printing. The STL files were subsequently sliced and converted to G-code for the printer *via* Slic3r software. Repetier Host v.2.1.1 was used as a user interface for controlling the 3D printer and printing parameters. During 3D printing, layer width was kept at 600 μm, layer height was set at 200 μm, and printing speed was 10 mm s^–1^. 3D printing was performed with a 410 μm tapered tip attached to the extruder. The hanging filament length, filament uniformity, printability quantifications, and filament collapse area of extruded filaments and 2D printed shapes were quantified using ImageJ.^43,44^ To determine filament uniformity, in each image of the 2D snaking lines, 15 thickness measurements were taken along each of the four long lines and compared to the specified thickness in the .stl files. To quantify printability, ImageJ was used to determine the perimeter and area of each square printed within the 2D grids. Equation 1 is used to calculate printability
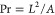
1where *L* is the perimeter and *A* is the area. To find the filament collapse remaining area, filaments were extruded onto a platform with seven columns that are separated by specifically designed gaps of 1, 2, 3, 4, 5, and 6 mm. Before the experiment, the area of each gap was recorded. After extrusion, the remaining area within each gap was compared to the initial area.

The 3D printing was carried out on a modified Anet A8 printer with a screw extruder-based printing head. The designs were generated in Solidworks 2019 and converted to STL files. The STL files were sliced and converted into G-code using Slic3r software. Repetier Host v.2.1.1 was used as a user interface for controlling the 3D printer. Layer height was kept at 200 μm, layer width was set at the inner diameter of the extruder tip, and printing speed was 5 mm s^–1^. 3D printing was performed with 410 or 1067 μm tapered extruder tapered tips attached to the extruder.

### 2.11 Loading and Release of FITC–BSA

Lyophilized CS-MA-ALD and CS-MA-ADH microgels were mixed at a 1:1 ratio to form eight samples, each with a mass of 5 mg. Each sample was placed into a separate centrifuge tube. To load FITC–BSA, 200 μL of a 100 μg/mL FITC–BSA solution was added to each tube. After 24 h at 4 °C, the samples were washed and centrifuged, and the supernatant was collected for loading efficiency determination. Half of the samples were submerged in 1 mL of PBS while the other half were submerged in 1 mL of 0.05 U/mL chondroitinase ABC. The samples were incubated at 37 °C, and at different time points, a small amount of PBS or enzyme was removed to perform fluorescent spectroscopy and evaluate the temporal release of FITC–BSA.

### 2.12 Cell Viability Assay and Cell Migration Assay

Fibroblasts (NIH/3T3, ATCC, USA) were cultured in normal growth media (DMEN, Gibco, USA), supplemented with 10% FBS (Life Technologies, USA) and 1% penicillin/streptomycin (100 U/100 μg/mL; Life Technologies, USA) at 37 °C with 5% CO_2_. For cell viability, 3T3 cells (10,000 cells) were co-cultured with the hydrogel samples, and the metabolic activities of the 3T3 cells were tested at 1, 3, and 5 days by alamarBlue (Bio-Rad) using 3T3 cells without the samples as the control group. For observing the cell adhesion behavior, 3T3 cells (1 × 10^6^ cells/ cm^2^) were seeded on top of cross-linked granular hydrogels with RGD modification (1 mM RGD, GCGYGRGDSPG, Genscript) through the thiol–ene reaction for 48 h. The cell-adhered hydrogels were further stained with Alexa Fluor 488 phalloidin (Thermo Fisher Scientific) and Hoechst (Thermo Fisher Scientific). Human umbilical vein endothelial cells (HUVECs, PELOBiotech GmbH) were infected with red-fluorescent protein (RFP)-expressing lentiviral particles at passage 1. RFP–HUVECs were cultured in endothelial cell growth medium-2 (EGM-2, Lonza) with 5% FBS prepared by supplementing the EBM-2 basal medium with the EGM-2 SingleQuots supplements from the EGM-2 endothelial cell growth medium-2 BulletKit (Lonza). Dynamic cross-linked granular hydrogels with 3–4 mm thickness were placed into a 40 ng/mL recombinant human platelet-derived growth factor-BB (PeproTech) overnight and then incubated in media overnight to remove unbounded PDGF-BB. RFP–HUVECs (5 × 10^5^ cells/cm^2^) were seeded on the top of the dynamically cross-linked granular hydrogels with and without PDGF-BB. Olympus FV3000 (Olympus Scientific Solutions Americas Corp., Waltham, MA) was used to image the distribution of the cells at 6 and 48 h. The invasion depth of individual cells was tracked by software provided by Imaris 9.9.1 (Oxford Instruments).

### 2.13 Statistical Analysis

All results are expressed as the mean ± deviation (*n* = 3–100). Statistical analysis of all quantitative data was performed *via* one-way ANOVA with post hoc Tukey tests or unpaired *t* test using GraphPad Prism (v 6.01).

## 3 Results and Discussion

### 3.1 Facile Synthesis and Fabrication of Granular Hydrogels

**Figure 1:**
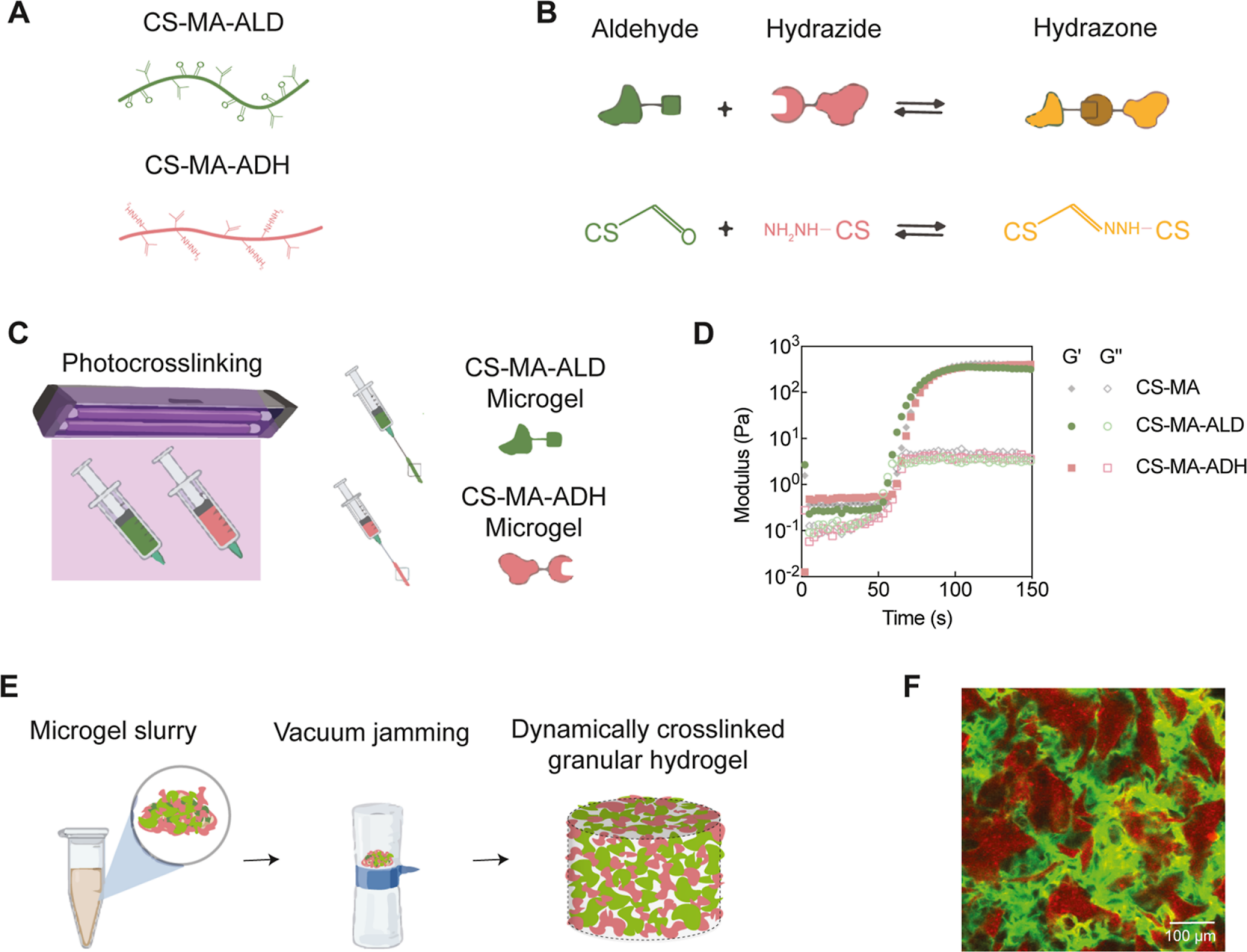
Fabrication of dynamic cross-linked granular hydrogels. (A) Chondroitin sulfate was dual-modified with methacrylate and aldehyde (CS-MA-ALD) or hydrazide (CS-MA-ADH). (B) Microgels formed by CS-MA-ALD and CS-MA-ADH were equipped with aldehyde groups and hydrazide groups, respectively, which could form interparticle dynamic covalent hydrazone bonds. (C) Microgels were produced by UV-cross-linking of precursor solutions followed by extrusion fragmentation of bulk hydrogels. (D) CS-MA, CS-MA-ALD, and CS-MA-ADH showed no significant difference in photo-cross-linking gelation kinetics. (E) CS-MA-ADH and CS-MA-ALD microgels were mixed with a 1:1 weight ratio to produce a microgel slurry. To ensure the formation of interparticle cross-linking, the slurry was jammed by vacuum filtration to produce dynamic cross-linked granular hydrogel. (F) Confocal images of dynamic cross-linked granular hydrogel (red: CS-MA-ADH microgels; and green: CS-MA-ALD microgels).

To begin fabricating granular hydrogels, methacrylate groups were first conjugated to CS to form CS-MA. CS-MA was subsequently modified with either aldehyde groups or hydrazide groups to generate CS-MA-ALD and CS-MA-ADH, respectively (Figures 1A, S1, and S2). The degree of methacrylation was determined to be ∼25% for each polymer. Hydrazide modification of CS-MA-ADH was determined to be ∼60%. The degree of aldehyde modification in CS-MA-ALD was determined to be ∼17%. Each of these values was determined using ^1^H NMR spectroscopy (Figures S3 and S4). Upon mixing, the aldehyde and hydrazide groups of microgels can form reversible hydrazone cross-linking of dynamic covalent bonds to improve the structural integrity while maintaining the injectability of granular hydrogels (Figure 1B).

There are three major methods available to form microgels, including microfluidics, batch emulsions, and extrusion fragmentation.^9^ Microfluidics and batch emulsion methods are useful for forming spherical gel particles with controlled sizes and relatively low polydispersity. The polydispersity is higher in microgels formed using batch emulsion than in microfluidics; however, both are significantly lower compared to microgels fabricated using extrusion fragmentation. While highly uniform particles are beneficial for some applications, microfluidic methods are time intensive and can be difficult to scale up. Moreover, since the size and polydispersity of batch emulsion gels are based on variables such as stir rate and rate of precursor addition, batch-to-batch variability is a challenge. In contrast, extrusion fragmentation is a simple, scalable, and highly repeatable method. Additionally, the irregularly shaped particles generated using extrusion fragmentation exhibit an increased surface area relative to other methods, leading to more interparticle contact and potentially increasing the formation of dynamic covalent cross-linking. For these reasons, extrusion fragmentation was chosen as the preferred method to fabricate granular hydrogels in this study.

First, the precursor solutions of bulk hydrogels were formulated by mixing 5 wt % of the CS-MA, CS-MA-ALD, or CS-MA-ADH polymers with 0.1 wt % photoinitiators. These hydrogel precursor solutions were cross-linked by UV light within a syringe and extruded through a series of needles to form irregularly shaped microgel fragments (Figure 1C). The gelation kinetics of the three types of CS polymers were similar, with the final storage modulus at ∼400 Pa (Figure 1D). To ensure similarity between different batches, the needle gauges and lengths were kept consistent throughout each synthesis. To form granular hydrogels, microgel slurries were jammed using vacuum filtration to facilitate packing (Figure 1E). In the dynamically cross-linked granular hydrogels, microgels of CS-MA-ALD and CS-MA-ADH were mixed at a 1:1 weight ratio. To ensure that this ratio was achieved, those microgels were lyophilized, mixed evenly in their dried state, rehydrated to 5 wt %, and then jammed. For non-cross-linked granular hydrogels, the CS-MA microgels were also jammed as a control group. By adding fluorescent dyes to the hydrogel precursor solutions, the microgels could be imaged using fluorescent microscopy to visualize the distribution of two types of microgels in the dynamically cross-linked granular hydrogels (Figure 1F).

### 3.2 Dynamically Cross-Linked Granular Hydrogels Display Both Mechanical and Structural Stability

**Figure 2:**
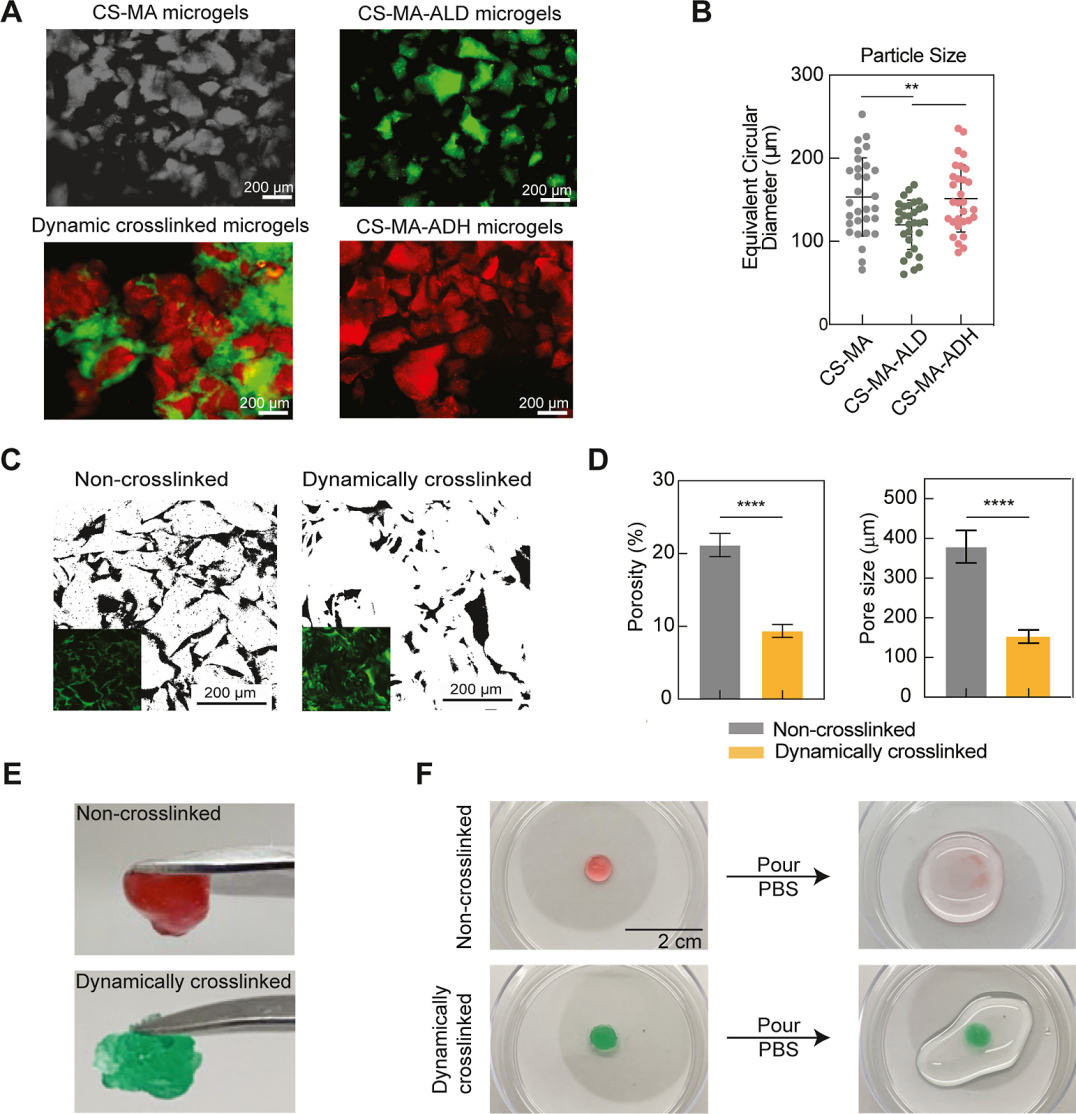
Morphologies of microgels and granular hydrogels. (A) Confocal images of CS-MA, CS-MA-ALD, and CS-MA-ADH microgels. The bonded microgel composites were observed in the dilution of the dynamically cross-linked granular hydrogel. (B) Equivalent circular diameters of CS-MA, CS-MA-ALD, and CS-MA-ADH microgels (*n* = 30). (C,D) FITC–dextran was infused into the matrix of non-cross-linked and dynamically cross-linked granular hydrogels to characterize their porosities and pore sizes (*n* = 26). (E,F) Morphology of non-cross-linked and dynamically cross-linked granular hydrogels. The dynamically cross-linked group remained integrated, but the non-cross-linked group dispersed after being poured with PBS. Statistics analysis was performed using a *t* test with **p < 0.01 and ****p < 0.0001.

To characterize the sizes of microgels, CS-MA and CS-MA-ALD microgels loaded with FTIC–dextran and CS-MA-ADH microgels loaded with rhodamine–BSA were observed with fluorescent microscopy (Figure 2A). By mixing the CS-MA-ALD and CS-MA-ADH microgels and pipetting the solution several times, the microgels were stuck together and formed aggregates as dynamically cross-linked microgels. It was determined that the CS-MA and CS-MA-ADH microgel fragments had average equivalent circular diameters of 153 ± 47 and 152 ± 40 μm, but the CS-MA-ALD microgels had a smaller average diameter of 119 ± 30 μm (Figure 2B). It was also noted that the CS-MA-ALD microgels swelled faster than the CS-MA-ADH microgels with the addition of PBS during the fragmentation process, potentially due to the reduction of the molecular weight of CS after oxidation. Therefore, the weaker mechanical strength of CS-MA-ALD hydrogels allowed the shear force to break the microgels into smaller fragments.

The porosity of granular hydrogels is an important characteristic that affects nutrient exchange and cell invasion. To measure the porosity of the granular hydrogels, the pore structures were visualized by swelling the granular hydrogels in a solution of fluorescent dye-tagged high-molecular-weight dextran (Figure 2C). The porosity and pore size in the dynamically covalent granular hydrogels decreased two-fold relative to the non-cross-linked granular hydrogels (Figure 2D). This decrease was likely due to increased interparticle cross-linking and the smaller sizes of CS-MA-ALD microgels, which reduced void space in the dynamic cross-linked granular hydrogels. As expected, the compact structure and interparticle cross-linking enhanced the mechanical stability of the dynamically cross-linked granular hydrogels. The non-cross-linked granular hydrogel was dispersed after being immersed in PBS, but the dynamically cross-linked granular hydrogels maintained structural integrity, indicating good stability of the cross-linked microgels for cell culture and hydrogel implantation (Figure 2E,F).

### 3.3 Dynamically Cross-Linked Granular Hydrogels Mechanically Recover after Extrusion

**Figure 3:**
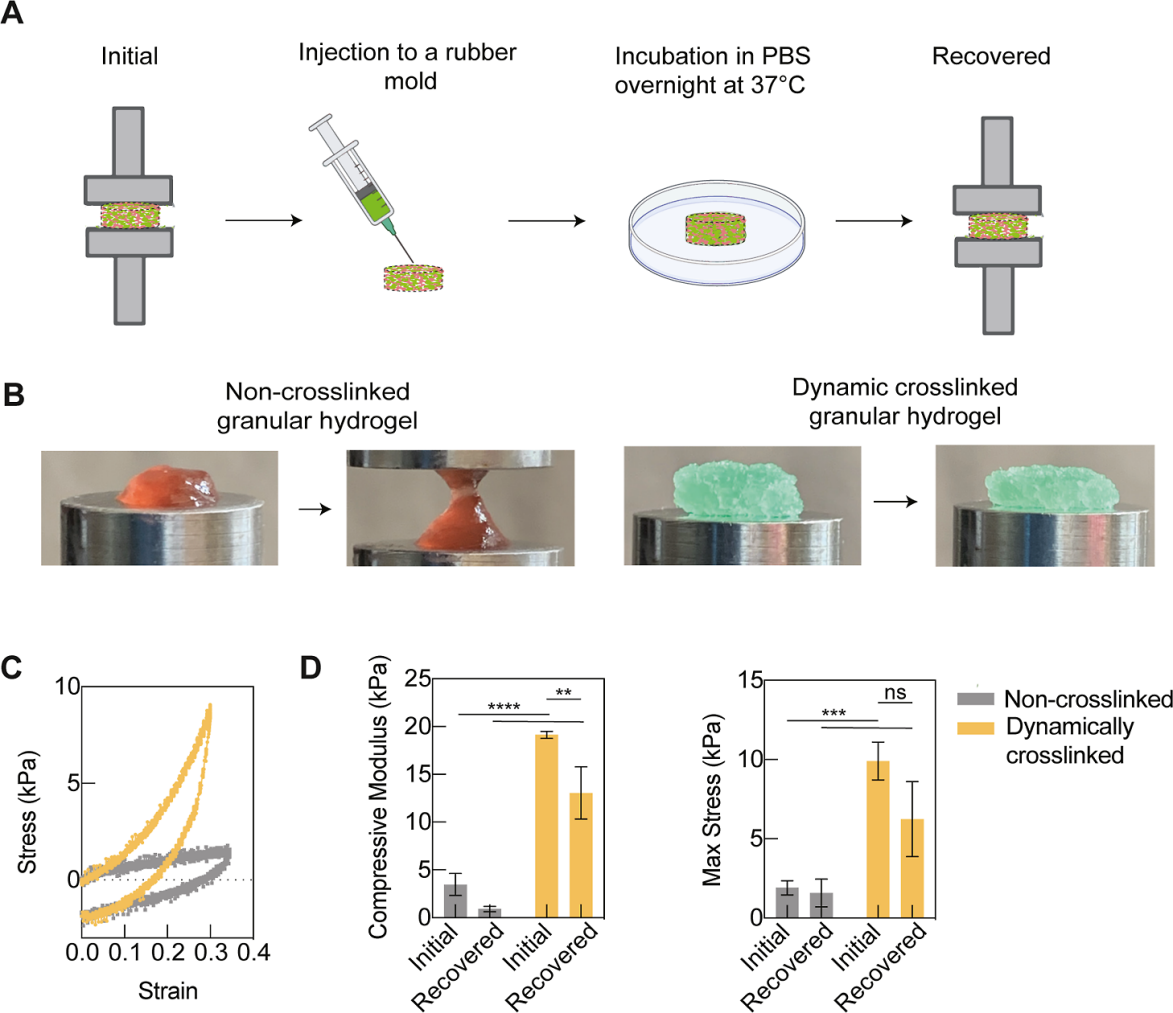
Compression tests were conducted on both non-cross-linked and dynamically cross-linked granular hydrogels to evaluate their interparticle cross-linking capabilities and self-healing properties. (A) Compression tests were performed on the non-cross-linked and dynamically cross-linked granular hydrogels before and after being injected into a rubber mold and incubated in PBS overnight at 37 °C to determine recovery. (B) Non-cross-linked group showed large deformation, but the dynamically cross-linked group showed only slight compression after the compression tests. (C) Stress–strain curves of the non-cross-linked and dynamically cross-linked granular hydrogels. (D) Compressive modulus and max stress of the dynamically cross-linked group were significantly higher than those of the non-cross-linked group, both initially and after recovery (*n* = 3). Statistics analysis was performed using one-way ANOVA with ns *p* > 0.05, **p < 0.01, ***p < 0.001, and ****p < 0.0001.

The mechanical stability of injectable granular hydrogels is crucial for 3D printing and therapeutic delivery. There are many post-injection methods to generate secondary cross-linking between particles for enhancing the mechanical strength of granular hydrogels, such as enzyme-mediated cross-linking and photo-cross-linking. However, these processes are often not suitable for deep tissue therapy or cause complications in the administration of granular hydrogels. Therefore, the mechanical properties were tested before and after injection to assess the mechanical stability of the dynamically cross-linked granular hydrogel and its suitability as an injectable hydrogel (Figure 3A). The mechanical properties of the granular hydrogels were measured through compression tests. Immediately after jamming, the non-cross-linked and dynamically cross-linked granular hydrogels were compressed to 30% thickness while recording the load applied. After this initial compression, the shape of the non-cross-linked samples was largely deformed, while that of the dynamically cross-linked samples resisted permanent deformation and remained as a cylinder (Figure 3B).

The stress–strain curves from the uniaxial compression test were employed to calculate the compression modulus and maximum stress values (Figure 3C). Due to the interparticle cross-linking through dynamic covalent bonds, the cross-linked granular hydrogels exhibited mechanical superiority to the non-cross-linked granular hydrogels. Initially, the compressive modulus and max stress of the cross-linked group increased greater than five-fold relative to the non-cross-linked group before injection. Moreover, the reversible nature of dynamic covalent bonds provides an additional attractive property to granular hydrogels in self-healing. In addition to their ability to detach in response to high strain, these bonds reform once the load is removed.

The extent of self-healing ability in the dynamic cross-linked granular hydrogels, as well as control samples, was analyzed quantitatively by assessing the recovery of the compressive modulus after injection. After assessing their initial compressive modulus, granular hydrogels were loaded into a syringe, injected into a PDMS mold, and incubated in PBS overnight at 37 °C. Upon removal from the mold, the compressive modulus of the recovered granular hydrogel was recorded and compared to the initial value (Figure 3D). The recovered dynamically cross-linked granular hydrogels were 68% of the initial modulus value, whereas the non-cross-linked granular hydrogels only recovered 26%. The compressive modulus and max stress of the cross-linked group at 13.05 ± 2.72 and 6.23 ± 2.36 Pa were also significantly higher than those of the non-cross-linked group at 0.90 ± 0.28 and 1.58 ± 0.87 Pa, respectively. Although the mechanical strength of dynamically cross-linked granular hydrogel could not completely recover after injection due to plastic deformation, these results confirm the improved self-healing properties and stability in PBS due to reversible cross-linking between the microgels.

### 3.4 Shear-Thinning and Self-Healing Characteristics of Granular Hydrogels Enable 3D Printing

**Figure 4:**
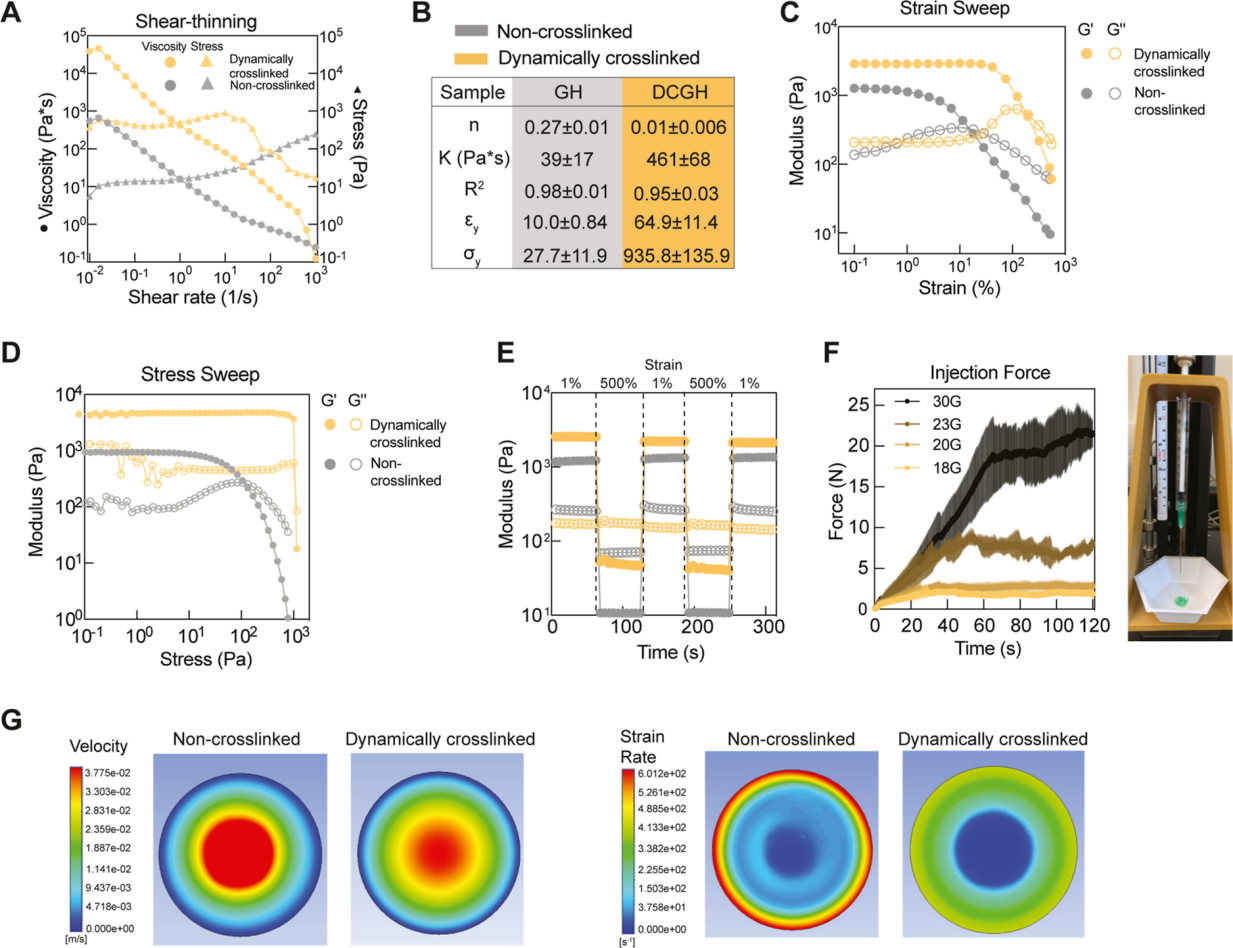
Rheological analysis and injection force tests of non-cross-linked and dynamically cross-linked granular hydrogels. (A) Shear-thinning behaviors of non-cross-linked and dynamically cross-linked granular hydrogels with the shear rate range from 0.01 to 1000 1/s. (B) Power law index (*n*) was calculated from fitting viscosity (η = *K*γ^*n*–1^) obtained from the shear-rate sweep, where *K* is the consistency factor and γ is the shear rate. The yield strains and stress of non-cross-linked and dynamically cross-linked granular hydrogels were measured by the (C) strain sweep (0.1–500% strain) and (D) stress sweep (0.1–1000 Pa), respectively (*n* = 3). (E) Self-healing ability of non-cross-linked and dynamically cross-linked granular hydrogels was assessed by applying alternating 1 and 500% strains. (F) Injection force needed to extrude the dynamically cross-linked granular hydrogels from a syringe with varying sizes of needles from 18 to 30G (*n* = 3). (G) Computational flow modeling for understanding the velocities and strain rates of non-cross-linked and dynamically cross-linked granular hydrogels during printing in a 22-gauge nozzle.

For therapeutic delivery and 3D printing, hydrogels should possess good injectability, shear-thinning, and self-healing ability. Jammed granular hydrogels have demonstrated their potential uses as injectable therapeutic carriers and 3D-printing inks.^7,23,39^ However, the low stability of granular hydrogels without interparticle cross-linking in a physiological environment limits their broader use. Granular microgels cross-linked by dynamic hydrazone bonds allowed it to be deformable and shear-thinning. The shear-thinning characteristics of the granular hydrogels were investigated by fitting the power law model to shear-rate sweep data. The power law index (*n*) is calculated from fitting viscosity (η = *K*γ^*n*–1^), where *K* is the consistency factor and γ is the shear rate. A Newtonian fluid will have *n* = 1, while a shear-thinning fluid will have 0 < *n* < 1. The dynamically cross-linked granular hydrogels had higher viscosity across all shear rates from 0.01 to 1000 s^–1^ than the non-cross-linked granular hydrogels due to the interparticle cross-linking. However, the viscosity of both groups decreased as the shear rate increased (Figure 4A,B), indicating shear-thinning behavior (*n* < 0.3) with power-law indexes at 0.27 and 0.01. To minimize the injection force required to deliver hydrogels through needles, the yield strain of injectable hydrogels should be as small as possible. However, if the yield strain is less than 50%, the structural integrity of the hydrogel may be compromised by tissue motion. The yield strain of the cross-linked group at 64.9 ± 11.3% was within the desired range for a low injection force and sufficient structural stability (Figure 4B,C). However, the yield strain of the non-cross-linked group at 10.0 ± 0.8% was too low to maintain structural stability after injection. Similarly, the yield stress of the dynamically cross-linked granular hydrogels was significantly improved relative to the non-cross-linked group (Figure 4D).

The dynamically cross-linked granular hydrogels also demonstrated structural integrity after vortexing in a centrifuge tube, whereas the non-cross-linked granular hydrogels became fluid-like due to the lack of connections between the microgels (Figure S5). Moreover, the reversible nature of hydrazone bonds allowed the dynamically cross-linked granular hydrogel to recover its elastic properties after experiencing large strains, ensuring the structural integrity of the hydrogel after injection. The rheological analysis of strain recovery was performed by applying alternating 1 and 500% of strains on both groups (Figure 4E). The recovery of the storage modulus at 1% strain indicated that both granular hydrogels possessed good self-healing ability under high strain. Finally, the shear-thinning and strain-yielding properties of the dynamically cross-linked granular hydrogels provide injectability by allowing the microgels to flow at high strain.

As multiple variables will affect the flow behavior of the granular hydrogels, it is important to also characterize their injectability for therapeutic delivery. In particular, there is a wide variety of needles (18G, 20G, 23G, and 30G) used in different clinical applications, and it is important to assess the feasibility of the granular hydrogel injection through these different needle gauges. Thus, to further assess injectability, the force required to inject samples through 1″ needles of varying bore diameter at 2 mL/h was measured (Figure 4F). The effect of needle length was also observed by comparing the injection through 20G needles of different lengths (1″ and 1 1/2″) (Figure S6).

The flow behavior of granular hydrogels was dependent on the ratio between the diameter of the flow channel (*D*) and the diameter of the particles (*d*). According to prior work, *D*/*d* ≤ 2.43 leads to minimal flow initially followed by clogging, while 2.43 < *D*/*d* < 5.26 results in intermittent bursting flow, and *D*/*d* ≥ 5.26 is required for continuous uniform flow.^45^ Based on the particle sizes of CS-MA-ADH and CS-MA-ALD microgels, *D*/*d* in the 18G, 20G, 23G, and 30G needles ranges from 2.14–5.66, 1.54–4.07, 0.86–2.28, and 0.41–1.07, respectively. It was determined that the dynamically cross-linked granular hydrogel shear-thinning properties alleviated clogging and bursting flow to an extent, with continuous flow through channels with *D*/*d* ≤ 2.43. With the 30G needle, a significantly larger force is required to extrude the dynamically cross-linked granular hydrogel. This indicates that as the needle’s inner diameter approaches the size of fragmented particles (*D*/*d* = 1), there is clogging and reduced flow. Additionally, injection through a 20G (1 1/2″) needle required a slightly increased force than the 20G (1″) at similar rates. This result was attributed to the increased needle length requiring extended extrusion of particles and, therefore, higher forces.

### 3.5 3D-Printed Mechanically Stable and Elastomeric Granular Hydrogels

**Figure 5:**
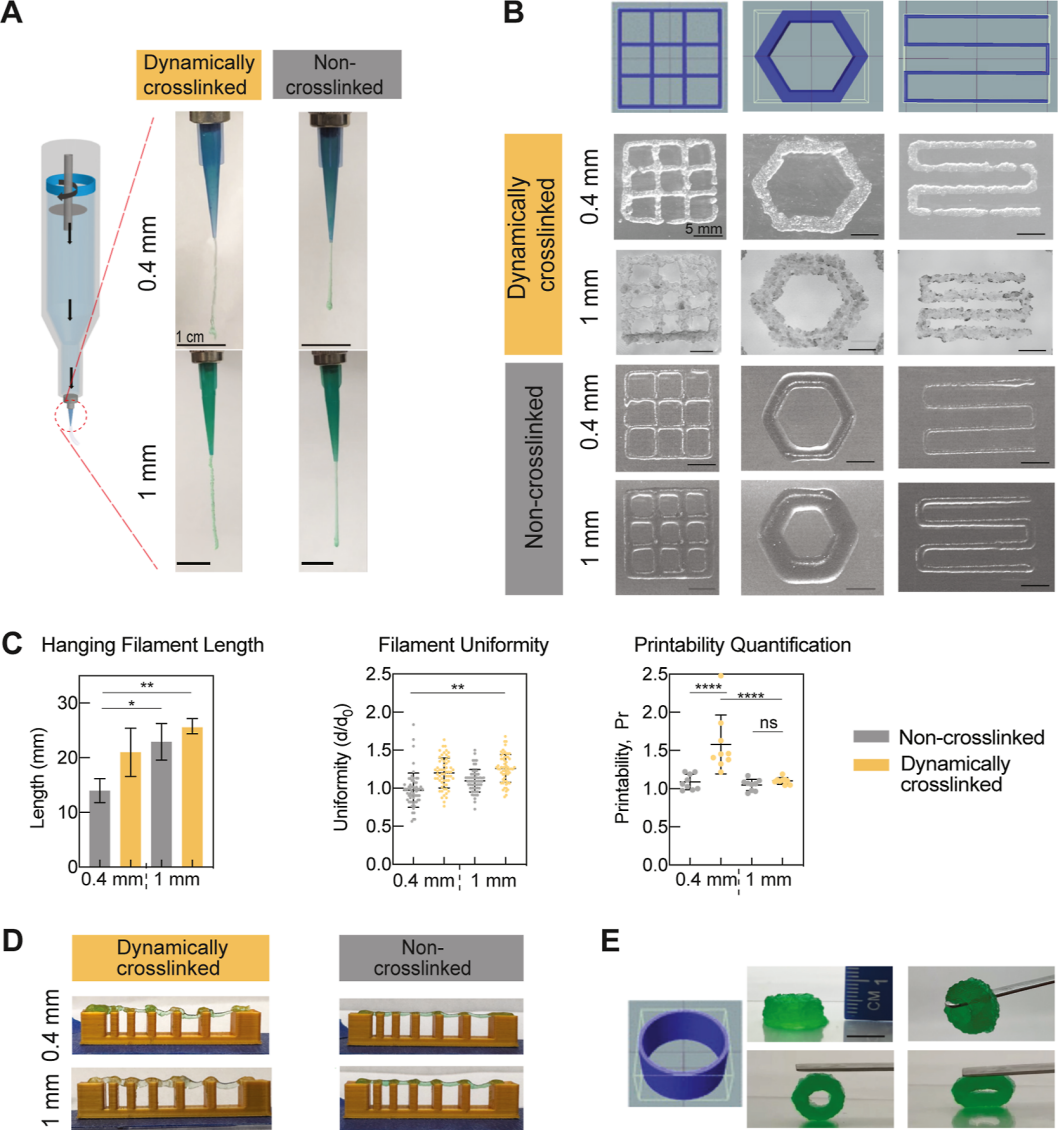
Shear-thinning and self-healing characteristics of granular hydrogels facilitate high print fidelity. (A) Image of hanging filaments formed using both the dynamically cross-linked and non-cross-linked microgels with both the 0.4 and 1 mm extruder tips. (B) Microgels were printed as grids, hexagons, and lines to investigate their printability using 0.4 and 1 mm nozzles (scale bar: 1 cm). (C) For different extrusion tips, the hanging filament length, filament diameter uniformity, and printability (geometric accuracy) were quantified. (D) Microgels were extruded across a platform containing seven columns, with specific gaps of 1, 2, 3, 4, 5, and 6 mm to assess print integrity by observing the extent to which filaments collapse within the gaps. (E) Dynamically cross-linked granular hydrogel was printed at a 0.5 mm height of the cylinder, which was mechanically stable without post-printing cross-linking (scale bar: 1 cm). Statistics analysis was performed using one-way ANOVA with ns *p* > 0.05, **p < 0.01, ***p < 0.001, and ****p < 0.0001.

Bioprinting is an emerging additive manufacturing approach to the fabrication of patient-specific, implantable 3D constructs for regenerative medicine. Extrusion bioprinting leverages shear-thinning and strain-yielding materials that are viscous at low shear, can flow at higher rates, and quickly stabilize after extrusion to form different structures. Some inks for extrusion printing require additional cross-linking after printing to ensure stability. However, the reversible dynamic covalent cross-links in the granular hydrogel provide self-healing properties that confer stability after extrusion without any additional cross-linking. The printability of dynamically cross-linked and non-cross-linked granular hydrogels was assessed by using shear-thinning characteristics acquired from the rheological tests to stimulate the velocities and strain rates of the granular hydrogels in the extrusion tip (Figure 4G). The results of the simulations show that the velocities and strain rates of dynamically cross-linked granular hydrogel became faster and decreased, respectively, from the center to the wall across the nozzle opening. This indicates that interparticle reversible cross-linking leads to plug flow behavior, and the ink moves as a viscoelastic solid while exiting the extruder tip. This behavior was demonstrated experimentally by performing hanging filament quantification, which showed that the extruded filament exhibited a length of >20 mm when using a 1 mm extrusion tip (Figure 5A). Dynamic cross-linking between microgel fragments leads to the formation of more stable filaments. This result further demonstrates shear-thinning and shear-recovery, indicating the potential of the dynamically cross-linked granular hydrogel to print 2D shapes and multilayer constructs.

The printability of the microgels was then assessed with basic shapes such as grids, hexagons, and lines (Figure 5B). Filament integrity was assessed by determining the extent of filament collapse across different gaps (Figure 5C). With shorter gaps (<1 cm), the cross-linked particles tend to clog and lead to over-extrusion, especially as the tip gets close to the platform surface. However, the cross-linking allows filaments to remain stable over larger gaps (Figure S8). When assessing filament uniformity using the printed 2D shapes, there was no significant difference between the different microgel groups at either tip size, which indicates that cross-linking does not reduce printability. Additionally, the printability (Pr) of the 2D shapes was quantified using eq 1. For squares, Pr = 1 indicates the highest level of dimensional accuracy. It is shown that the geometric accuracy of printed structures was slightly improved when using 1 mm extrusion tips relative to 0.4 mm, especially in the dynamic cross-linked microgels (Figure 5D). This result is similar to the injection force measurements with varying gauges of needles as a higher extrusion force required for 0.4 mm tips might cause burst extrusion and reduce fidelity. To illustrate the practical utility of dynamically cross-linked granular hydrogel for tissue defects, we demonstrate how to create full-scale bioprinted implants with good mechanical stability. Finally, the dynamically cross-linked granular hydrogel was printed as a 5 mm cylinder tube with 50 layers, which demonstrated elasticity and mechanical stability without post-printing cross-linking (Figure 5E).

### 3.6 Granular Hydrogels Promote Sustained Release of Protein Therapeutics

CS is known for its ability to form protein complexes by electrostatic force. Zeta-potential analysis was performed on each of the polymers (Figure S9). It was determined that CS-MA and CS-MA-ALD were slightly more negatively charged than CS-MA-ADH due to the positive charge of the hydrazide groups. However, the negative charge of all the polymers suggests that the granular hydrogels can achieve efficient loading and controlled release of cationic biomolecules. Granular hydrogels, as stimuli-responsive injectable biomaterials, have applications in tissue regeneration, tumor therapy, and genetic engineering. In each of these applications, granular hydrogels are loaded with a form of therapeutic cargo to deliver and achieve a certain effect. For this reason, the ability of the dynamic covalent granular hydrogels to load FITC–BSA and perform controlled release was assessed.

**Figure 6:**
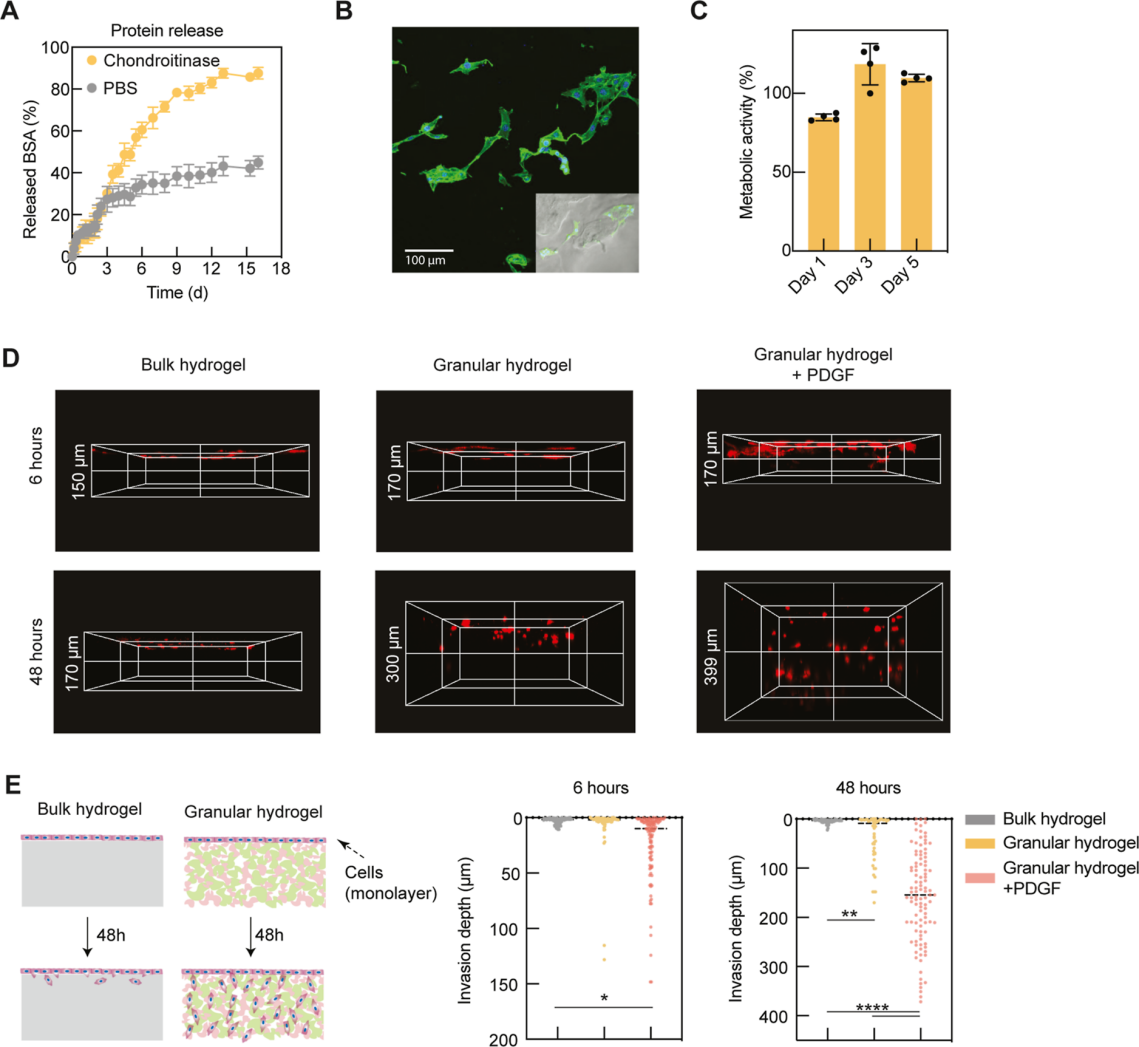
Dynamically cross-linked granular hydrogel facilitates the sustained release of therapeutic to promote cell migration. (A) Release profiles of a model protein (BSA) from the granular hydrogels in PBS with or without 0.05 U/mL chondroitinase ABC (*n* = 3). (B) Cell adhesion morphology on RGD-functionalized dynamically cross-linked granular hydrogels (scale bar: 100 μm, green: actin; and blue: nuclear). (C) Metabolic activity of the cells co-cultured with the granular hydrogels at day 1, 3, and 5 days (*n* = 4, control: TCPS). (D) Confocal images and (E) quantification of cell migration and invasion on the CS-MA bulk hydrogels, granular hydrogels, and PDFG-BB-loaded granular hydrogels at 6 and 48 h after seeding (*n* = 60). Statistics analysis was performed using one-way ANOVA with ns *p* > 0.05, **p < 0.01, ***p < 0.001, and ****p < 0.0001.

To determine the release rate under stable physiological conditions and assess the potential impact of enzymatic activity, the release in 0.05 U/mL chondroitinase ABC was compared to that in PBS (Figure 6A). The granular hydrogels showed a high loading efficiency of FITC–BSA (72.4 ± 2.6%) and a limited burst release, with only ∼13% release after 2 days in both PBS and enzyme. For the first 3 days, the release rates in the enzyme and PBS were nearly identical. However, from day 3 to 11, the fractional release in enzyme increased to 80.3 ± 2.44%, while release in PBS only increased to 38.9 ± 3.97% over the same period. This delayed increase in the release rate was assumed to be the result of an initial diffusion-dependent release mechanism followed by a more significant influence of granular hydrogel dissolution in the later stages. The interparticle cross-linking remained stable for the initial 3 days, imitating bulk degradation. However, fragmented microgel particles slowly dissolved due to the activity of chondroitinase. To further understand the mechanism of protein release, the data were fit to mathematical models of therapeutic delivery (Figure S10). For the release in PBS, the power law model was initially chosen as this is a general equation used to describe release dependent on diffusion and relaxation (eq 2).
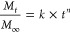
2In the power law model, *n* is the diffusional component of release. When *n* = 0.5, this indicates release solely based on diffusion, while *n* = 1 describes relaxation-dependent release. For the granular hydrogels in PBS, *n* = 0.54, which indicates release that is mainly driven by diffusion (*R*^2^ = 0.949). Enzymatic conditions led to a significant increase in the rate of release, showing more time-dependent behavior. Therefore, the data were fit to the equation for zero-order drug release (*R*^2^ = 0.957) (eq 3).
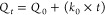
3

In PBS, the granular hydrogels resist degradation and perform a controlled release of loaded therapeutics. The diffusion-driven bulk degradation of the granular hydrogels is slow and begins to plateau after 6 days. However, enzymatic conditions lead to at least a 3-fold increase in degradation and release rates (Figure S11). Therefore, the dynamically cross-linked granular hydrogel can be used as an enzyme-responsive injectable hydrogel for therapeutic delivery.

### 3.7 Release of Protein Therapeutics from Granular Hydrogels Support Cell Proliferation and Migration

Granular hydrogels can support the repair of injured tissues by guiding local cell behavior.^46^ The porosity, polymer compositions, and growth factors released from granular hydrogels could present crucial signals for the recruitment of cells. Modulating cell invasion in tissue scaffolds is a central tenet of endogenous tissue repairs, such as cell invasion, migration, proliferation, and matrix deposition. First, to promote cell adhesion to the dynamically cross-linked granular hydrogels, the precursor solutions of microgels were supplemented with 1 mM RGD. After 48 h, the seeded 3T3 cells showed good cell adhesion behavior on the hydrogel matrix, which is an initial and important step for cell invasion (Figure 6B). To assess the cytocompatibility of the hydrogels, alamarBlue was used to evaluate the metabolic activity of co-cultured 3T3 cells. The metabolic activities of cells co-cultured with the hydrogel samples on days 1, 3, and 5 were all above 80% compared to the control, indicating good cytocompatibility (Figure 6C).

To test the potential of dynamically cross-linked granular hydrogels for therapeutic delivery and tissue repair, HUVECs were seeded on bulk hydrogels, granular hydrogels, and PDGF-BB-loaded granular hydrogels (Figure 6D). PDGF-BB is a key angiogenic protein able to stabilize newly formed blood vessels during angiogenesis. Previous research has demonstrated that hydrogels containing PDGF-BB can stimulate HUVEC migration, subsequently attracting endogenous host endothelial cells to promote tissue growth.^38,47−49^ At 6 h, no obvious cell invasion was observed in the bulk hydrogels or granular hydrogels, and slight cell invasion was seen in the PDGF-BB-loaded granular hydrogels. At 48 h, cell invasion into the granular hydrogels was significantly higher compared to the bulk hydrogels, and cell invasion was further enhanced in the PDGF-BB-loaded granular hydrogels. This result indicates that the loaded PDGF-BB and porosity of granular hydrogels had a synergistic effect on HUVEC recruitment (Figure 6E). The PDGF-BB-loaded dynamically cross-linked granular hydrogels have shown the potential of an injectable tissue engineering scaffold that could present physical and biochemical signals to drive endogenous repair. Other growth factors with high affinity to CS, such as TGF-β and bone morphogenetic protein 2 (BMP-2), could be further incorporated into this granular hydrogel for tissue engineering applications.

## 4 Conclusions

Chondroitin sulfate microgels functionalized with aldehyde or hydrazide groups were produced by a simple fragmentation process and used to fabricate granular hydrogels with hydrazone dynamic covalent cross-linking. These materials exhibited good injectability, sheer thinning, and self-healing ability. In addition, the dynamic interparticle cross-linking significantly improved the mechanical stability and strength of the granular hydrogels after injection, which is beneficial for retaining the microgels at injury sites. The injectability was assessed by rheological analysis and injection force measurements with varying diameters of needles. Although the dynamically cross-linked granular hydrogel had higher viscosity and yield strain than un-cross-linked granular hydrogels, it still possessed suitable injectability for applications like 3D printing and therapeutic delivery. Utility for extrusion-based 3D printing was demonstrated by printing into different shapes and showing good print fidelity. Finally, the electrostatic charge of chondroitin sulfate was exploited for growth factor loading and sustained release, which enhanced cell invasion. The presence of porosity, cell adhesion peptides, and growth factors on microgels could modulate cell behaviors. Overall, the dynamically cross-linked granular hydrogel can be used as an injectable and printable scaffold for tissue engineering and regenerative medicine.

## Supporting Information Available

Additional data related to chemical modifications of chondroitin sulfate, NMR spectra, inversion test for network stability, injection force as a function of needle length, stability of extruded filaments, zeta-potential analysis, mathematical modeling of release data, degradation experiment, and visual demonstration of pore interconnectivity (PDF)
