# Supplementary material for: Dynamically Cross-Linked Granular Hydrogels for 3D Printing and Therapeutic Delivery
Source: ACS Appl Bio Mater. 2023 Aug 16;6(9):3683–95. doi: 10.1021/acsabm.3c00337 (PMC10863386; doi:10.1021/acsabm.3c00337)
Supplement: Supplementary file 1 — mt3c00337_si_001.pdf [file mt3c00337_si_001.pdf]

Supporting Information

Dynamically Crosslinked Granular Hydrogels for 3D Printing and Therapeutic Delivery

Hung-Pang Lee[1†], Ryan Davis, Jr.[1†], Ting-Ching Wang[2], Kaivalya A. Deo[1], Kathy Xiao Cai[1], Daniel L. Alge[1,3], Tanmay P. Lele[1,2], Akhilesh K. Gaharwar[1,3,4,5,]*

[1] Biomedical Engineering, College of Engineering, Texas A&M University, College Station, Texas 77843, USA

[2] Chemical Engineering, College of Engineering, Texas A&M University, College Station, Texas 77843, USA

[3] Material Science and Engineering, College of Engineering, Texas A&M University, College Station, Texas 77843, USA

[4] Interdisciplinary Graduate Program in Genetics & Genomics, Texas A&M University, College Station, Texas 77843, USA.

[5] Center for Remote Health Technologies and Systems, Texas A&M University, College Station, Texas 77843, USA

[†] These authors contributed equally to this work.

[*]Corresponding author: gaharwar@tamu.edu (A.K.G.)

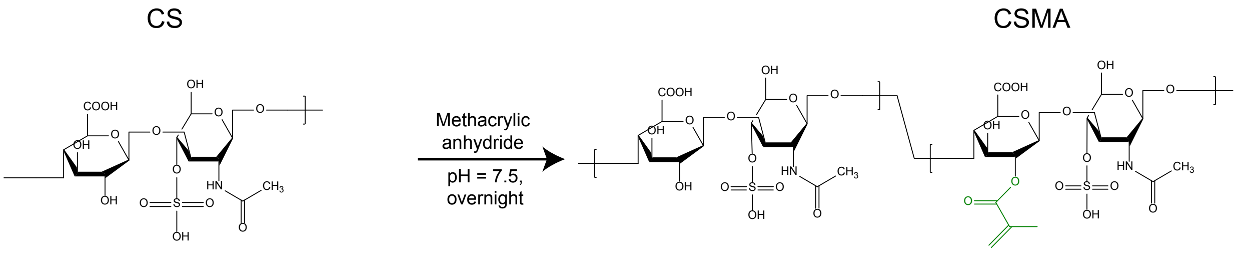

Figure S1. The synthesis of methacrylated chondroitin sulfate (CSMA).

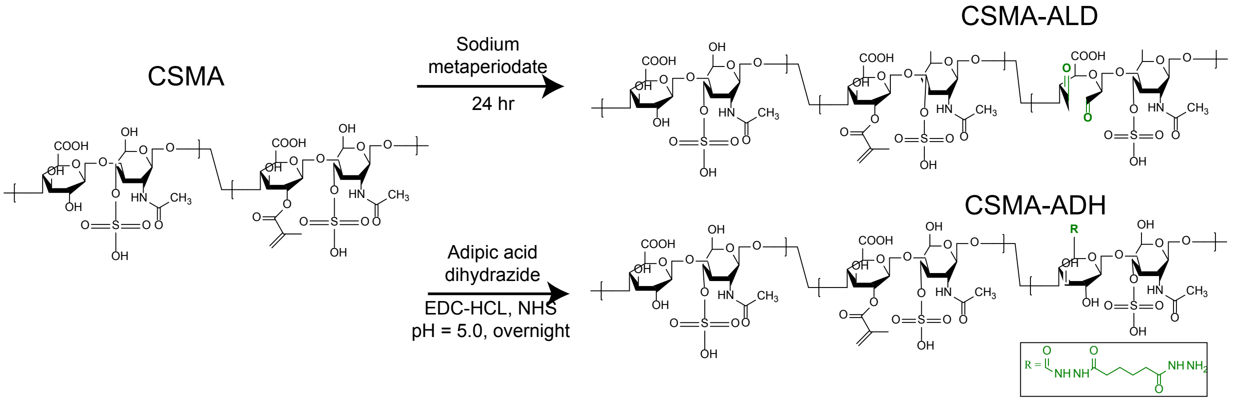

Figure S2. The modification of CSMA with aldehyde groups (CSMA-ALD) and hydrazide groups (CSMA-ADH).

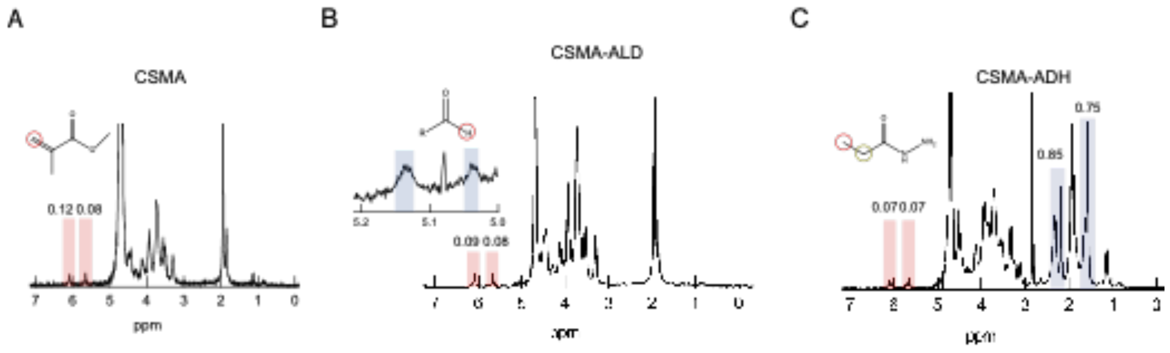

Figure S3. The ^{1}H-NMR spectra of CSMA, CSMA-ALD, CSMA-ADH A) Methacrylate-modified chondroitin sulfate (CSMA, ~30% degree of methacrylate modification). B) Methacrylate- and aldehyde-modified chondroitin sulfate (CSMA-ALD, ~25.5% degree of methacrylate modification) and degree of aldehyde modification was further characterized by grafting tert-butyl carbazate (TBC) to CSMA-ALD through reductive amination. C) Methacrylate- and hydrazide-modified chondroitin sulfate (CSMA-ADH, ~21% degree of methacrylate modification, and ~60% degree of hydrazide modification). Functional group peaks are normalized to the CS backbone and quantified by peak integration.

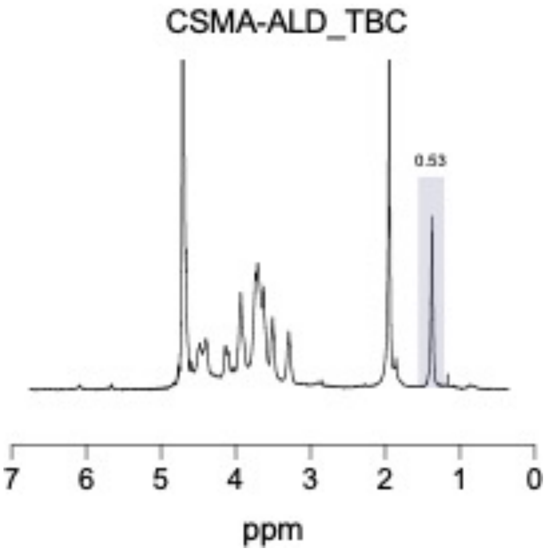

Figure S4. The aldehyde group peaks of CSMA-ALD could be quantified directly. Therefore, tert-butyl carbazate (TBC) was introduced to modify aldehyde groups to quantify the aldehyde degree of CSMA-ALD (~17% degree of aldehyde modification).

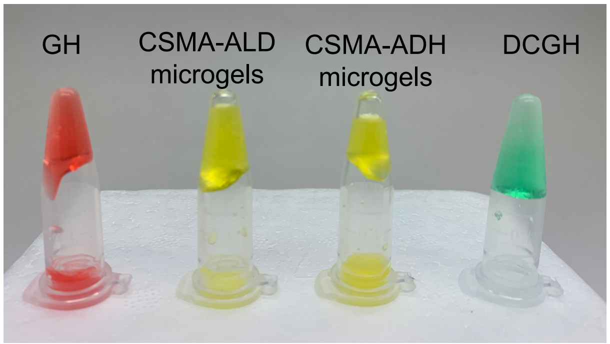

Figure S5. Dynamic crosslinked granular hydrogel remained stable in the centrifuge tube after being vortexed, but granular hydrogel, CSMA-ALD microgels, and CSMA-ADH microgels flowed when the tubes were inverted.

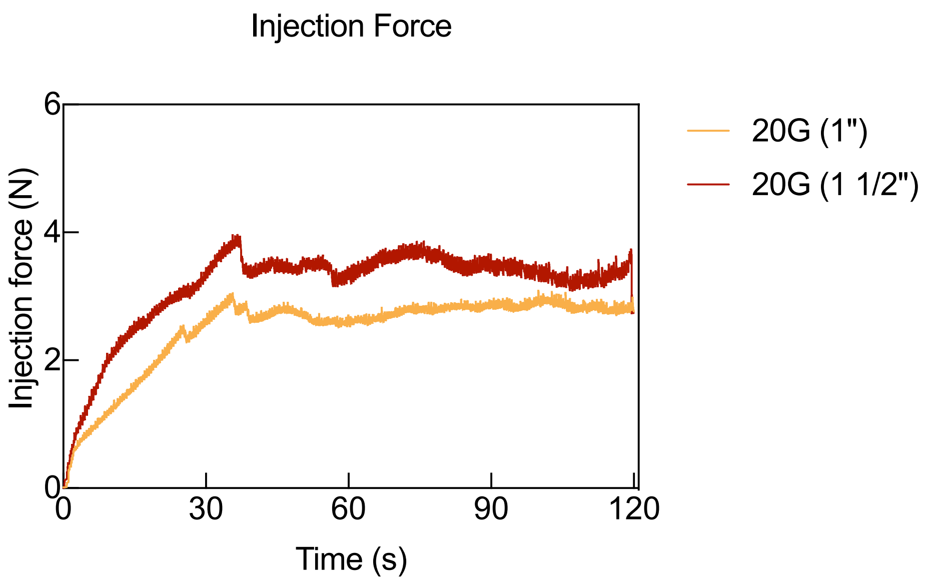

Figure S6. The injection force to inject DCGH using 20 G with 1” and 1 1/2” lengths. Longer needle would also increase the injection force.

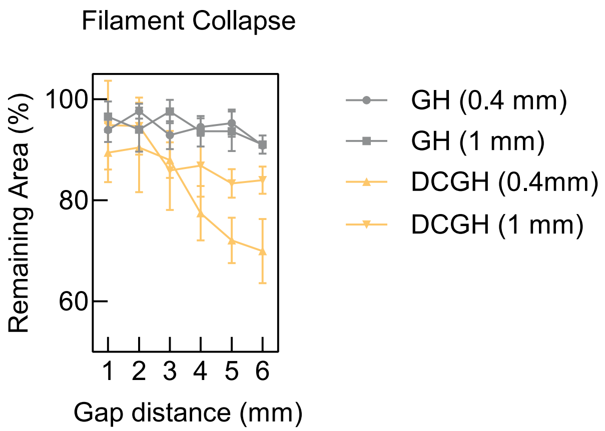

Figure S7. The remaining area after filament collapse was quantified for the non-crosslinked and dynamically crosslinked microgels using both the 0.4mm and 1mm tips.

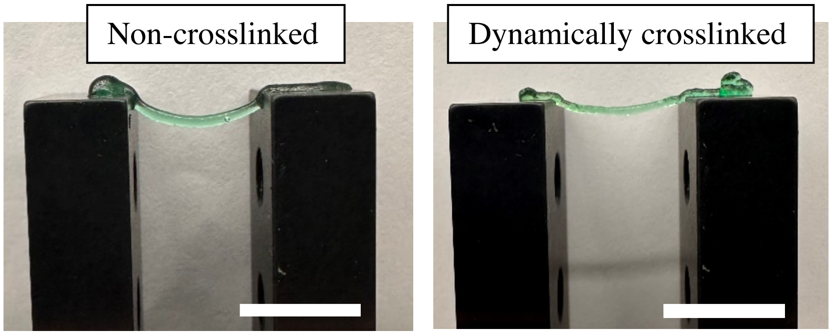

Figure S8. When extruded over a >1cm gap, dynamically crosslinked microgels show improved stability relative to non-crosslinked microgels.

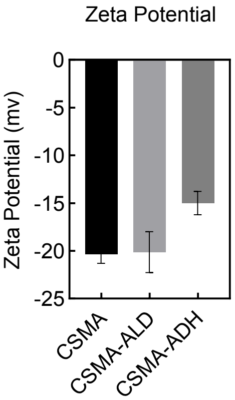

Figure S9. Zeta potential of CSMA, CSMA-ALD, and CSMA-ADH. CSMA-ADH has slightly higher zeta potential than CSMA and CSMA-ALD due to the positive charge of hydrazide.

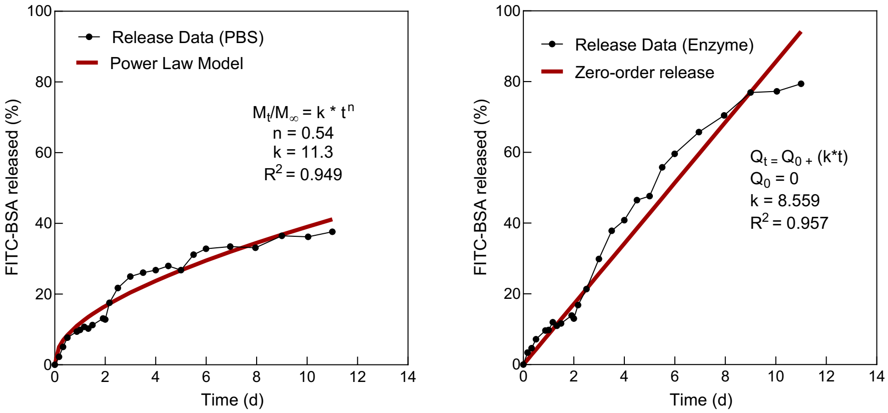

Figure S10. Fitting of FITC-BSA release data to mathematical models of drug release. In PBS, the release data fit to the power law model with $R^2 = 0.949$. In enzymatic conditions, the release fit to the zero-order release model with $R^2 = 0.957$.

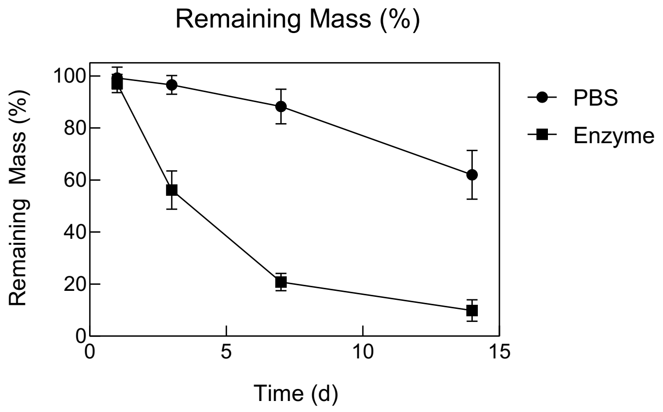

Figure S11. Degradation of dynamic covalent granular hydrogels in PBS and 0.05 U/mL chondroitinase ABC. Granular hydrogels maintained nearly 60% mass over 14 days in PBS, while enzymatic conditions led to over 90% degradation within the same period.

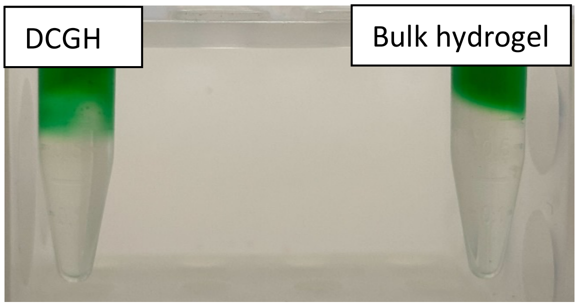

Figure S12. Visual demonstration of pore interconnectivity. After 5 min, dyed PBS is able to diffuse further through the dynamic crosslinked granular hydrogel.
